# Antibacterial Activity against Foodborne Pathogens and Inhibitory Effect on Anti-Inflammatory Mediators’ Production of Brazilin-Enriched Extract from *Caesalpinia sappan* Linn

**DOI:** 10.3390/plants11131698

**Published:** 2022-06-27

**Authors:** Thanawat Pattananandecha, Sutasinee Apichai, Jakaphun Julsrigival, Fumihiko Ogata, Naohito Kawasaki, Chalermpong Saenjum

**Affiliations:** 1 Center of Excellence for Innovation in Analytical Science and Technology for Biodiversity-Based Economic and Society (I-ANALY-S-T_B.BES-CMU), Chiang Mai University, Chiang Mai 50200, Thailand; thanawat.pdecha@gmail.com (T.P.); sutasinee.apichai@gmail.com (S.A.); jakaphun@gmail.com (J.J.); 2 Department of Pharmaceutical Sciences, Faculty of Pharmacy, Chiang Mai University, Chiang Mai 50200, Thailand; 3 Faculty of Pharmacy, Kindai University, 3-4-1 Kowakae, Higashiosaka 577-8502, Japan; ogata@phar.kindai.ac.jp (F.O.); kawasaki@phar.kindai.ac.jp (N.K.); 4 Antiaging Center, Kindai University, 3-4-1 Kowakae, Higashiosaka 577-8502, Japan

**Keywords:** *Caesalpinia sappan* L., brazilin, anti-inflammatory, antibacterial, foodborne pathogen

## Abstract

*Caesalpinia sappan* L. heartwood was collected from Mae Chaem District, Chiang Mai Province, Thailand. Crude extracts were prepared by Soxhlet’s extraction using 50, 60, and 70% of ethanol (EtOH) at 50, 60, and 70 °C, and the brazilin content was measured using reversed-phase high performance liquid chromatography (RP-HPLC). The antibacterial activity against foodborne pathogens and anti-inflammatory aspects were investigated. *C. sappan*, prepared from 70% EtOH at 70 °C (E70T70), significantly (*p* < 0.05) exhibited the highest amount of brazilin (7.90 ± 0.50% *w*/*w*). All extracts were investigated for anti-inflammatory activity through an inhibition effect on nitric oxide (NO) and inducible nitric oxide synthase (iNOS) production in RAW264.7 mouse macrophage cells. The inhibitory effect on cyclooxygenase-2 (COX-2) production in HT-29 and HCT116 was also studied. All the extracts inhibited NO, iNOS, and COX-2 production induced by combined lipopolysaccharide and interferon-γ, especially E70T70, indicating the highest inhibition effect among other extracts. Additionally, E70T70 was selected to determine the antibacterial activity against foodborne pathogens, including *Staphylococcus aureus*, *Escherichia coli*, *Salmonella enteritidis*, and *Vibrio parahaemolyticus*. The result showed that 200 µg/mL extract reduced all test pathogens 100% at 24 h. These results suggested the potential of using *C. sappan* L. extract as a natural preservative in food and a natural active pharmaceutical ingredient.

## 1. Introduction

Foodborne diseases are a major public health concern worldwide, with significant implications for travel, commerce, and development [1]. Bacteria are the most prevalent cause of foodborne diseases, and they come in a wide range of forms, types, and properties [2], including *Salmonella* spp., *Bacillus cereus*, *Escherichia coli*, *Staphylococcus aureus*, *Clostridium botulinum*, *Clostridium perfringens*, *Vibrio* spp., *Cronobacter sakazakii*, *Listeria monocytogenes*, and *Shigella* spp. [2,3,4]. Foodborne illness occurs when a pathogen is consumed with food and establishes itself; the illness is known as foodborne infection or foodborne intoxication [2]. Antibiotics are routinely used to treat human and animal infections [5,6,7]. The overuse and misuse of antibiotics in veterinary medicine have become a serious issue in recent times [8,9]. One of the most serious implications of antibiotic residues in animal diets is the spread of antibiotic-resistant microorganisms that are harmful to human health. Antibiotic-resistant pathogenic bacteria in food might cause difficult-to-treat foodborne infections among humans. They can also transfer antibiotic-resistant genes (ARGs) onto other microbes via the food production chain [5,10]. The rise of multidrug-resistant bacterial pathogens from many sources, particularly in the food chain, increases the need for effective antimicrobial agent use in both livestock and healthcare systems [5,7,10].

*Caesalpinia sappan* L. is a species of the Leguminosae family, also known as Sappan wood and Indian redwood [11]. It is a native plant in Southeast Asia and distributed across Africa and the Americas [12]. *C. sappan* dried wood has been used as a traditional ingredient in food and beverages [13], its extract has shown preservative properties [14] and was found to be nontoxic in rats [15]. *C. sappan* has a wide range of biological activity, including antioxidant, anti-inflammatory, antibacterial, anti-acne, hypoglycemic, hepatoprotective and vasorelaxation activity, immunomodulation, and xanthine oxidase inhibition [11,16]. *C. sappan* heartwood has been used as a traditional food and beverage ingredient [13], traditional skincare [17], folk medicine [15], and natural coloring agent [16]. *C. sappan* heartwood and wood contain various kinds of flavonoids and phenolic compounds including brazilin, brazilein, caesalpin J, brazilide A, protosappanin A, protosappanin B, protosappanin E, neosappanone A, caesalpin P, sappanchalcone, 3-deoxysappanone, 4-*O*-methylsappanol, xanthone, and coumarin [11,16,17,18]. Brazilin (7,11*b*-dihydrobenz [*b*] indeno [1,2-*d*]pyran-3,6a,9,10(6H)-tetrol) is one of *C. sappan* main active compounds. Brazilin has been reported to exert antioxidant, anti-inflammatory, and antibacterial activities [11], and shows antibacterial activity against antibiotic-resistant bacteria, including methicillin-resistant *Staphylococcus aureus* (MRSA), vancomycin-resistant enterococci (VRE), and multidrug resistant *Burkholderia cepacia* with the minimal inhibitory concentrations (MIC) ranging from 4 to 32 µg/mL [19].

Therefore, this study aims to prepare and standardize brazilin-rich extract (BRE) from *C. sappan* heartwood and investigate their antibacterial against the foodborne pathogens, including *Staphylococcus aureus*, *Escherichia coli*, *Salmonella enteritidis*, and *Vibrio parahaemolyticus* and anti-inflammatory effect on nitric oxide (NO), inducible nitric oxide synthase (iNOS) and cyclooxygenase-2 (COX-2) in combined lipopolysaccharide (LPS) and interferon-γ (IFN-γ)-stimulated colon adenocarcinoma and carcinoma cells. The BRE extract with potent antibacterial and anti-inflammatory activities might be used as a natural preservative in food and a natural active pharmaceutical ingredient (NAPI) to prevent foodborne pathogens colonization and their related inflammation symptoms in functional food and nutraceutical products.

## 2. Results and Discussion

### 2.1. Determination of Brazilin Content

**Figure 1:**
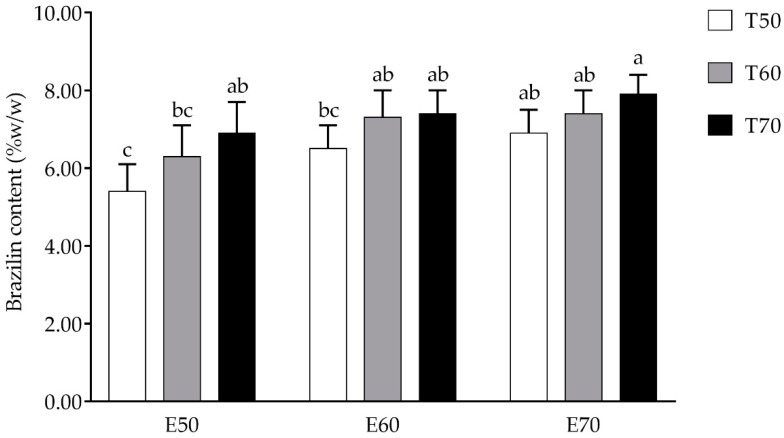
Brazilin content of *C. sappan* L. extract samples. Different superscript letters indicate a significant difference at *p* < 0.05.

**Figure 2:**
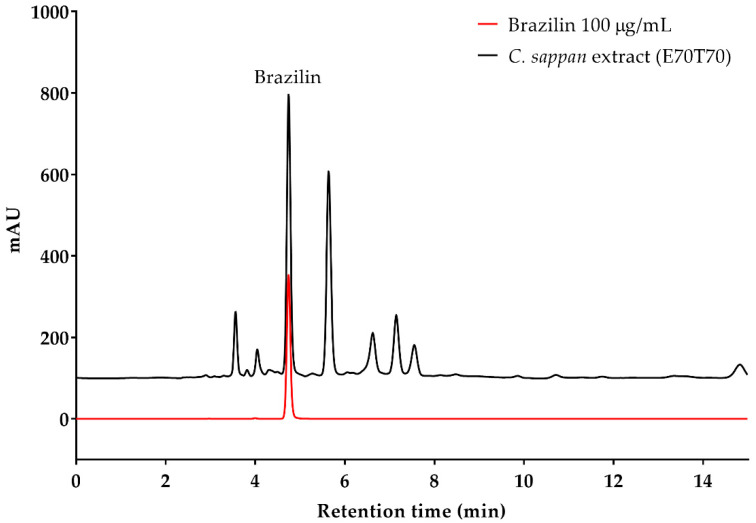
HPLC chromatogram of standard brazilin and *C. sappan* L. extract sample (E70T70).

The ethanol extracts of *C. sappan* L. contained brazilin ranging from 5.40 to 7.90% *w*/*w*. Crude *C. sappan* L. extract prepared with 70% ethanol at 70 °C (E70T70) exhibited the highest (*p* < 0.05) brazilin content at 7.90 ± 0.50% (*w*/*w*), while the extract prepared with 50% ethanol at 50 °C (E50T50) exhibited the lowest content of brazilin (*p* < 0.05) as shown in Figure 1. The HPLC chromatogram of the standard brazilin and *C. sappan* L. extract sample (E70T70) is shown in Figure 2, which demonstrates that brazilin has a retention time of 4.68 min.

Brazilin has been reported as the major compound in *C. sappan* L. Brazilin is lipophilic with a K_*a*_ of 5.2 and Log P of +1.3 [20] and has a higher affinity for organic solvents [21,22,23]. In this study, the percentage of ethanol and temperature affected the brazilin content extracted from *C. sappan*. In this case, adding ethanol ratio reduces the polarity of the solvent [24]. Ethanolic extract of *C. sappan* L. heartwood contained brazilin at 1.259 ± 0.285 and 1.256 ± 0.266 g/100 g of dried heartwood (TLC-densitometry and TLC-image analysis, respectively) [25]. *C. sappan* heartwood samples collected from various locations in Thailand produced methanolic extract using Soxhlet’s extraction. The brazilin content in the extract samples ranged from 8.7 to 22.2% (*w*/*w* of the extract) analyzed by high performance liquid chromatography (HPLC) [21]. Furthermore, a recent study by Hwang and Shim [23] reported that 20 µg/mL of water extract *C. sappan* L. contained 1.74–4.4 µg/mL of brazilin. When exposed to high temperatures, plant tissues soften, and weak interactions alter cell membranes. Consequently, phenolic compounds may be extracted into the solvent with relative ease [26]. Furthermore, the increasing extraction temperature may increase the solubility of brazilin as the relationship between solubility and temperature was exponential [27]. Moreover, the variation of brazilin content in the extract is also due to pulverizing and extraction time, the age of the tree, and geographic differences [20].

### 2.2. Determining Anti-Inflammatory Activities

The IC_50_ values of the extract samples concerning the inhibition effects on nitric oxide, iNOS, and COX-2 production are shown in Table 1. All *C. sappan* L. extracts at 50 µg/mL significantly inhibited (*p* < 0.05) NO and iNOS production in RAW264.7 cells without exerting cytotoxicity. Additionally, all extract samples also inhibited COX-2 production in HT-29 and HCT116 cells induced by combined LPS and IFN-γ. The *C. sappan* L. extract prepared with 70% EtOH at 70 °C significantly exhibited the highest potent inhibitory effect on NO, iNOS, and COX-2 production (*p* < 0.05), among other extracts. Remarkably, a correlation was found between the brazilin content of the extracts and the production of each inflammatory cytokine, as shown in Table S1.

Inflammation is a defensive reaction to trauma, infection, toxic compounds, irradiation or tissue injury [28]. During the inflammation process, macrophages play a key role in managing various immune-pathological conditions, including overproducing pro-inflammatory cytokines and inflammatory mediators such as NO, iNOS, COX-2, and tumor necrosis factor alpha (TNF-α) [29,30]. The anti-inflammatory effect of *C. sappen* L. extracts in this study was consistent with related research [29,31,32,33]. The methanolic extract of *C. sappan* L. was reported to inhibit COX-2 and iNOS expression in RAW 264.7 macrophage cells [31]. Moreover, brazilin exhibited anti-inflammatory activity via various mechanisms, including inhibiting NO production and regulating nuclear factor kappa-B (NF-κB) and activator protein-1 in RAW 264.7 cells [29,32,33].

### 2.3. Antimicrobial Activity of C. sappan L. Extracts

**Figure 3:**
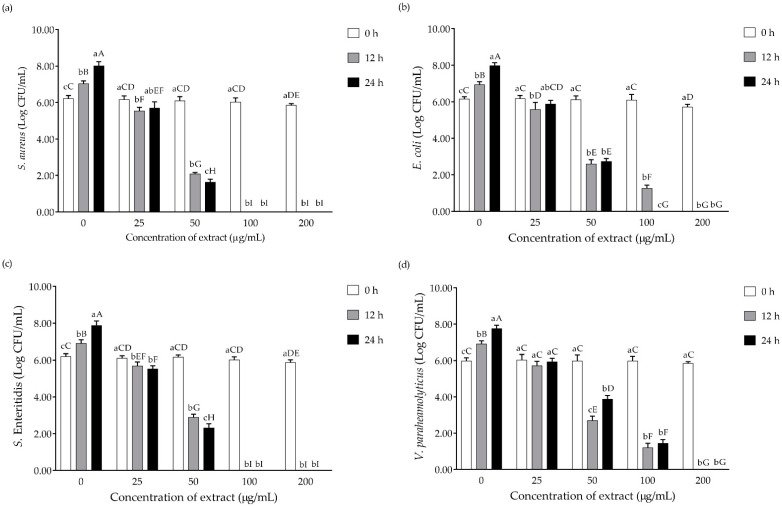
Antimicrobial activity of *C. sappan* L. extracts against foodborne pathogens (**a**) *S. aureus*, (**b**) *E. coli*, (**c**) *S. enteritidis*, and (**d**) *V. parahaemolyticus*. The values are expressed as mean ± SD (*n* = 3). Different superscript letters indicate a significant difference at *p* < 0.05. The lowercase letters indicate the significant difference between each incubation time of the same concentration of the samples, and capital letters indicate the significant difference between all samples.

Antibiotic resistance is a significant global health issue requiring immediate concern and the development of novel antibacterial medicines [34]. Natural products derived from plants and microbes have historically offered a wealth of valuable chemicals leading to the development of novel medications and therapies [35,36]. The extract prepared from E70T70 was selected to determine its antimicrobial activity against foodborne bacterial strains, including *S. aureus*, *E. coli*, *S. enteritidis*, and *V. parahaemolyticus*. The viable cell count was expressed as log CFU/mL of the tested pathogens. The *C. sappan* extract (E70T70) was selected to determine the antimicrobial activity against foodborne bacterial strains according to the highest amount of brazilin and potent anti-inflammatory activities as described previously. The cultured cell number was compared between the bacteria cultivated with each concentration of *C. sappan* extract (E70T70) and all the concentrations on different culture times, as shown in Figure 3. The antimicrobial activity was related to the concentrations of *C. sappan* extract (E70T70). Reduced test pathogens appeared in the presence of *C. sappan* L. extract (E70T70), even at the lowest concentration of 25 µg/mL. *C. sappan* L. extracts (E70T70) at 100 and 200 µg/mL significantly reduced (*p* < 0.05) the number of *S. aureus* and *S. enteritidis* and reduced 100% at 24 h of the incubation period, while *C. sappan* L. extracts at 200 µg/mL significantly reduced (*p* < 0.05) the number of *E. coli* and *V. parahaemolyticus* at 100% at 24 h of the incubation period. 

*S. aureus*, *E. coli*, *Salmonella*, *Shigella*, and *Vibrio* were identified as the most prevalent foodborne pathogens [37,38,39]. However, the prevalence rates could be related to the difference in time, location, and research season [37]. The sensitivity and resistance of pathogens against antimicrobial agents depend on the bacteria’s strain and source [40]. The cell membrane structure of bacteria is related to the different resistance levels to antibiotics, dyes, detergents, and disinfectants [40,41]. Gram-positive bacteria have thicker outer peptidoglycan layers that absorb antibiotics and disinfectants more readily, making them easier to kill. In contrast, Gram-negative bacteria are immune to some physical assaults because they do not absorb foreign materials surrounding them [42]. Plants’ antibacterial activities are linked to their ability to produce several secondary metabolites with antimicrobial properties that are relatively complex structures [43]. The antibacterial activity of plant extracts is dependent not only on the presence of secondary metabolites but also on their concentration and possible interactions with other components [43,44]. As a flavonoid, brazilin’s antibacterial activity is due to its ability to bind with intracellular and soluble proteins and bind with bacterial cell walls, then it exert its action by causing leakage [45]. The result indicated that *C. sappan* L. extracts (E70T70) have antimicrobial ability by inhibiting the tested foodborne pathogens, including *S. aureus*, *E. coli*, *S. enteritidis*, and *V. parahaemolyticus*. Especially *S. aureus*, which is a Gram-positive bacteria, is the most sensitive bacteria against *C. sappan* extracts (E70T70). At the same time, *S. enteritidis* was the weakest Gram-negative bacteria among *E. coli* and *V. parahaemolyticus*. Currently, there is a lack of data on the comparative study of brazilin’s antibacterial activity against the Gram-negative bacteria; however, the sensitivity of Gram-negative bacteria could be related to the variation of the outer membrane properties of strain [42,46]. Brazilin showed antibacterial activity against antibiotic-resistant bacteria, including MRSA, VRE, and multi-drug resistant *Burkholderia cepacia,* with an MIC ranging from 4 to 32 µg/mL [19]. Various solvents were used to extract *C. sappan* heartwood, and the methanolic extract of *C. sappan* extract showing higher antimicrobial activity against methicillin-sensitive *S. aureus* (MSSA) and MRSA than n-butanol, chloroform, and water extracts [47]. Ethanolic extracts of *C. sappan* also exerted antimicrobial activity against *Pseudomonas aeruginosa*, *S. aureus*, *S. typhi*, *Enterobacter aerogens*, *Candida albicans, and E. coli* with a maximum inhibition zone of 34, 31, 24, 21, 20, 15, and 14 mm, respectively [48]. Moreover, brazilin from *C. sappan* exerted an inhibitory effect on *Propionibacterium acnes*. Methanol and 50% ethanol extracts of *C. sappan* exhibited the highest inhibitory activity on *P. acnes* compared with another 39 medicinal plants [49]. Brazilin also showed a higher inhibitory effect on *P. acnes* than protosappanin A and sappanone B found in methanolic *C. sappan* extracts [17].

## 3. Materials and Methods

### 3.1. Chemicals and Reagents

Brazilin and curcumin were obtained from Sigma Chemical Co. (St. Louis, MO, USA). All chemicals and solvents used were standard, analytical or HPLC grade. They were purchased commercially from Sigma Chemical Co., Ltd. (St. Louis, MO, USA), Merck Co., Ltd. (Kenilworth, NJ, USA), or Fluka Chemical Co. (Buchs, Switzerland). All chemicals and reagents used in the cell-based study were purchased from Invitrogen (Waltham, MA, USA) and Roche (Mannheim, Germany).

### 3.2. Extracting C. sappan L.

*C. sappan* L. heartwood was collected from Mae Chaem District, Chiang Mai Province, Thailand. *C. sappan* L. heartwood sample was dried at 50 °C in a hot air oven for 24 h and then ground into a powder using a grinder machine. Crude extracts were prepared by Soxhlet’s extraction at 50, 60, and 70% ethanol (E50, E60 and E70, respectively) at the ratio of dried *C. sappan* L. and ethanol at 1:10 for 50, 60, and 70 °C (T50, T60 and T70, respectively) for 180 min. The solutions were then filtered using Whatman filter paper (No. 1) and followed by evaporating under reduced pressure until concentrated and dried in the vacuum dryer. The extracts were kept at −20 °C until use.

### 3.3. Determining Brazilin Content Using Reversed-Phase HPLC

The *C. sappan* L. heartwood extracts were measured for brazilin content using reverse phase HPLC with the modified method of Yan-yan et al. [50]. The HPLC uses an Agilent 1200 equipped with a multi-wavelength detector. The detection wavelength was set at 280 nm. The assay was carried out using a Kinetex^®^ polar C18 column (4.6 mm × 150 mm, 2.6 µm particle diameters, Phenomenex Co., Ltd., Torrance, CA, USA) and 0.1% acetic acid in methanol and deionized water in the ratio of 25:75 was used for the mobile phase at the flow rate of 0.6 mL/min. The column temperature was set to 25 °C with an injected sample volume of 10 µL.

### 3.4. Determining Anti-Inflammatory Activities

#### 3.4.1. Inhibitory Effect on Nitric Oxide (NO) and Inducible Nitric Oxide Synthase (iNOS) Production in RAW264.7 Cells

The inhibition effect on NO and iNOS production in RAW 264.7 cells was determined using the method from our related study [51]. Briefly, the cells were cultured in 24-well plates using Dulbecco’s modified Eagle’s medium (DMEM) supplemented with 10% fetal bovine serum (FBS), 100 units/mL penicillin and 100 µg/mL streptomycin. Then cells were maintained at 37 °C and 5% CO_2_ for 12 h. The cultured cells were then removed and replaced with a fresh medium containing the extract samples. After 12 h of incubation, the cells were activated with LPS (2 ng/mL) and IFN-γ (50 pg/mL) and incubated at 37 °C with 5% CO_2_ for 72 h. The NO production was measured in the supernatants of the cultured medium using the Griess reagent at 540 nm spectrophotometrically. The nitrite concentrations were calculated from a standard curve of potassium nitrite. The iNOS production in cell lysates was determined using a commercially available mouse iNOS ELISA Kit (CSB-E08326M, Cusabio Biotech, Co., Ltd., Houston, TX, USA). CelLytic^TM^ M Cell Lysis Buffer (Sigma, C2978) was used to prepare the cell lysate, and curcumin was used as the positive control. Cell viability and protein concentrations of control samples and those stimulated with combined LPS and IFN-γ for 72 h were assayed using cell viability reagent (PrestoBlue^TM^, Invitrogen, Waltham, MA, USA) and Bradford reagent, respectively.

#### 3.4.2. Inhibitory Effect on Cyclooxygenase-2 (COX-2) Production in HT-29 and HCT 116 Colorectal Cells

HT-29 and HCT116 colorectal cells were pre-incubated in 24-well plates for 24 h. Then the media was removed and replaced with fresh medium containing the samples. After 12 h of incubation, the cells were activated with LPS (2 ng/mL) and IFN-γ (50 pg/mL) and incubated at 37 °C with 5% CO_2_ for 72 h. Then the cells were lysed to yield cell lysates using CelLyticTM M Cell Lysis Buffer (Sigma, C2978) to determine COX-2 production using a commercially available human COX-2 ELISA Kit. Curcumin was used as the positive control. The protein produced by HT-29 and HCT116 colorectal cells was analyzed using the Bradford reagent (Sigma Chemical Co., Ltd., St. Louis, MO, USA). Concurrently, the viability of HT-29 and HCT116 colorectal cells with and without activation with combined LPS-IFN-γ was assayed according to the improved methods reported by Sirithunyalug et al. [52], in the absence or presence of samples for 72 h using the cell proliferation reagent WST-1 (Roche, Basel, Switzerland).

### 3.5. Determining Antimicrobial Activity against Foodborne Pathogens

The *C. sappan* L. extract was evaluated for antimicrobial activity against foodborne pathogens, including *S. aureus* ATCC 25923, *E. coli* ATCC 25922, *S. enteritidis* ATCC 14028, and *V. parahaemolyticus* ATCC 17802. The determination was performed using the slightly modified method of Sirilun et al. [53]. Briefly, a total of 200 µL of each strain inoculum was mixed with the different concentrations of the tested sample (1800 µL). The final concentrations of the tested samples were 25, 50, 100, and 200 µg/mL. The negative control was a mixture of medium broth and tested samples without any tested pathogens, while the positive control was established by combining medium broth and each tested pathogen. All tested tubes were incubated by shaking at 180 rpm at 37 °C for 0, 12, and 24 h. Then the viable cells were comparably counted on tryptic soy agar (TSA) at 24 h, whereas the viable cells for *V. parahaemolyticus* were counted on TSA supplemented with 3% NaCl.

### 3.6. Statistical Analysis

SPSS Software (version 17.00) was used to analyze all the data statistically. One-way ANOVA was used to test any significant difference between treatments, *p* < 0.05 was considered significant, and further significance between groups was analyzed using a Duncan post hoc test. Results are presented as the mean ± standard deviation of three independent experiments.

## 4. Conclusions

*C. sappan* extract prepared with 70% EtOH at 70 °C had the highest amount of brazilin and also showed the potential to reduce the growth of foodborne pathogens with 200 µg/L at 24 h of incubation. Moreover, the extract also showed an inhibitory effect on proinflammatory mediators, NO and iNOS in RAW264.7 and COX-2 in HT-29 and HCT116 cells. These results suggested that *C. sappan* L. extract shows potential to be used as a natural preservative in food and natural pharmaceutical active ingredients due to its antibacterial and anti-inflammation properties.

## Figures and Tables

**Table 1:** IC_50_ on inhibition effect on nitric oxide, iNOS, and COX-2 production.

Samples/ Positive Control	IC_50_ (ppm)			
	NO	iNOS	COX-2 (HT-29)	COX-2 (HCT 116)
E50T50	37.49 ± 1.27 ^a^	40.15 ± 1.36 ^a^	43.28 ± 1.38 ^a^	47.75 ± 1.47 ^a^
E50T60	32.62 ± 1.36 ^b^	37.33 ± 1.39 ^b^	37.75 ± 1.45 ^b^	43.52 ± 1.38 ^b^
E50T70	25.53 ± 1.44 ^d^	25.35 ± 1.40 ^c^	34.18 ± 1.27 ^c^	37.25 ± 1.39 ^c^
E60T50	30.07 ± 1.37 ^c^	35.19 ± 1.42 ^b^	38.18 ± 1.35 ^b^	41.59 ± 1.42 ^b^
E60T60	22.45 ± 1.19 ^e^	24.82 ± 1.28 ^c^	27.64 ± 1.23 ^d^	28.63 ± 1.32 ^d^
E60T70	19.54 ± 1.28 ^f, g^	20.95 ± 1.32 ^de^	28.22 ± 1.13 ^d^	29.92 ± 1.47 ^d^
E70T50	24.74 ± 1.33 ^d^	24.74 ± 1.33 ^c^	32.88 ± 1.27 ^c^	35.43 ± 1.18 ^c^
E70T60	21.37 ± 1.25 ^e, f^	22.41 ± 1.19 ^d^	27.65 ± 1.22 ^d^	28.65 ± 1.258 ^d^
E70T70	17.68 ± 1.32 ^g^	19.84 ± 1.26 ^e^	24.32 ± 1.37 ^e^	26.18 ± 1.25 ^e^
Curcumin	12.72 ± 1.13 ^h^	14.29 ± 1.19 ^f^	17.11 ± 1.02 ^f^	19.38 ± 1.19 ^f^

All values are expressed as mean ± standard deviation (*n =* 3). Different letters in each tested method indicate a significant difference (*p* < 0.05).

## Data Availability

The original contributions generated for this study are included in the article; the data presented in this study are available on request from the corresponding author.

## Supplementary Materials

The following supporting information can be downloaded at: https://www.mdpi.com/article/10.3390/plants11131698/s1, Table S1: Correlation coefficients (r^2^) between assay for brazilin extracts.
