# Supplementary material for: Antibacterial Activity against Foodborne Pathogens and Inhibitory Effect on Anti-Inflammatory Mediators’ Production of Brazilin-Enriched Extract from Caesalpinia sappan Linn
Source: Plants (Basel). 2022 Jun 27;11(13):1698. doi: 10.3390/plants11131698 (PMC9269513; doi:10.3390/plants11131698)
Supplement: Supplementary file 1 [file plants-11-01698-s001.zip › plants-1786722-supplementary.pdf]

Table S1. Correlation coefficients (r^2) between assay for brazilin extracts[18].

		NO	iNOS	COX-2 (HT-29)	COX-2 (HCT116)
Brazilin	Pearson Correlation	-0.664**	-0.645**	-0.654**	-0.664**
	Sig. (2-tailed)	0.000	0.000	0.000	0.000
	N	27	27	27	27

**. Correlation is significant at the 0.01 level (2-tailed).
